# Supplementary figures and images for: The etiology of attention deficit disorder with hyperactivity: A protocol for an umbrella review
Source: PLoS One. 2025 Jan 24;20(1):e0318141. doi: 10.1371/journal.pone.0318141 (PMC11759348; doi:10.1371/journal.pone.0318141)

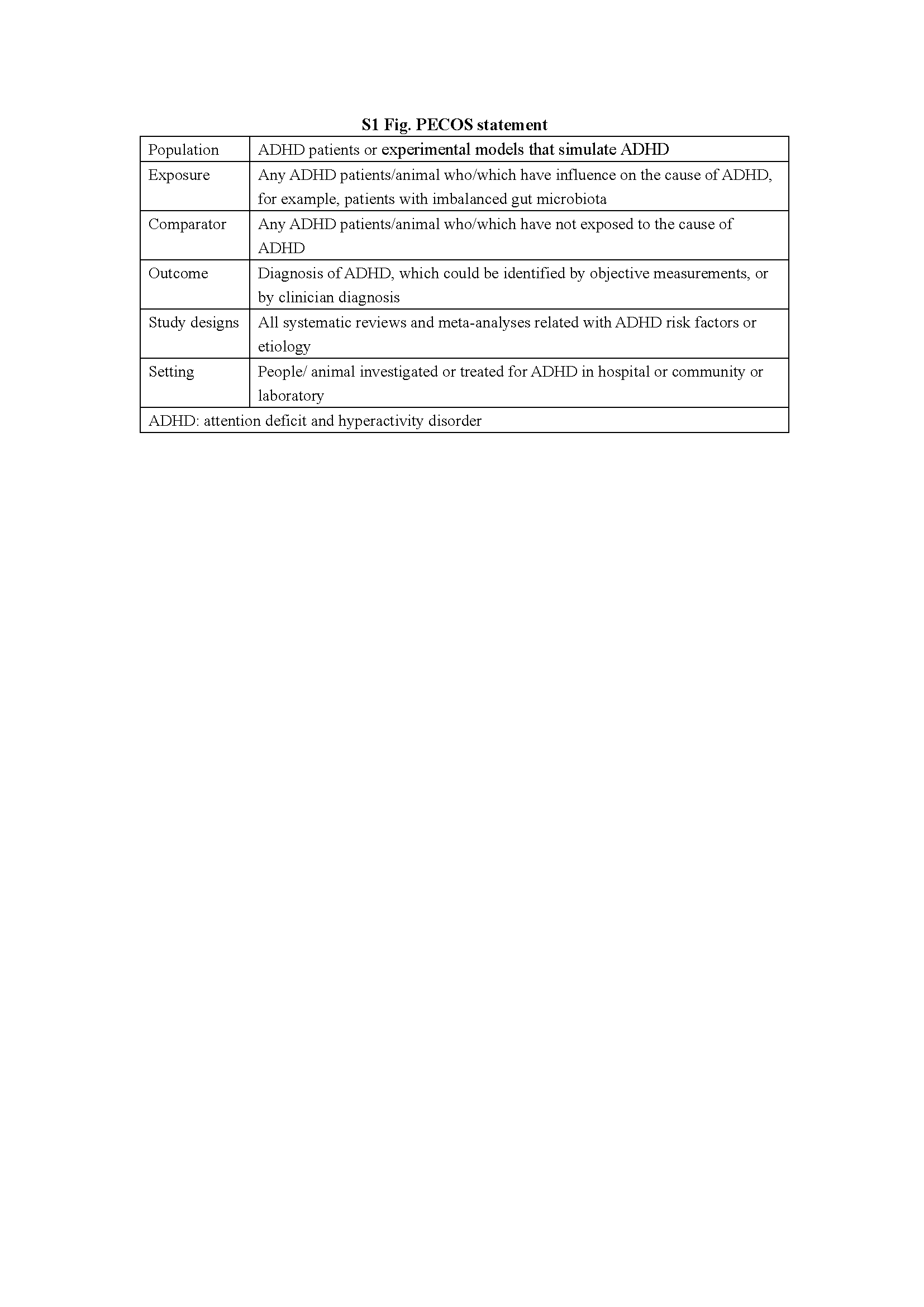

Supplement: S1 Fig — (TIF) [file pone.0318141.s001.tif]
