# Supplementary material for: The etiology of attention deficit disorder with hyperactivity: A protocol for an umbrella review
Source: PLoS One. 2025 Jan 24;20(1):e0318141. doi: 10.1371/journal.pone.0318141 (PMC11759348; doi:10.1371/journal.pone.0318141)
Supplement: S2 File — (PDF) [file pone.0318141.s003.pdf]

S2 File Search strategy

PubMed		
**Search**	**Query**	**results**
#1	"Attention Deficit Disorder with Hyperactivity"[MeSH Terms] OR "ADHD" [All Fields] OR "attention deficit hyperactivity disorder" [All Fields] OR "behavior disorder" [All Fields] OR "ADDH" [All Fields] OR "Attention Deficit Disorders with Hyperactivity" [All Fields] OR "Attention Deficit Hyperactivity Disorders" [All Fields] OR "Attention Deficit Hyperactivity Disorder" [All Fields] OR "Attention Deficit-Hyperactivity Disorder" [All Fields] OR "Attention Deficit-Hyperactivity Disorders" [All Fields] OR "Deficit-Hyperactivity Disorder, Attention" [All Fields] OR "Deficit-Hyperactivity Disorders, Attention" [All Fields] OR "Disorder, Attention Deficit-Hyperactivity" [All Fields] OR "Disorders, Attention Deficit-Hyperactivity" [All Fields] OR "Hyperkinetic Syndrome" [All Fields] OR "Syndromes, Hyperkinetic" [All Fields] OR "Attention Deficit Disorder" [All Fields] OR "Attention Deficit Disorders" [All Fields] OR "Deficit Disorder, Attention" [All Fields] OR "Deficit Disorders, Attention" [All Fields] OR "Disorder, Attention Deficit" [All Fields] OR "Disorders, Attention Deficit" [All Fields] OR "Brain Dysfunction, Minimal" [All Fields] OR "Dysfunction, Minimal Brain" [All Fields] OR "Minimal Brain Dysfunction" [All Fields]	
#2	"Risk Factors"[MeSH Terms] OR "epidemiologic factors"[MeSH Terms] OR "risk factor*"[All Fields] OR "risk variable*" [All Fields] OR "predictor*"[All Fields] OR "prediction" [All Fields] OR "predict* factor*" [All Fields] OR "prognostic factor*"[All Fields] OR "prognostic variable*" [All Fields] OR "epidemiologic factor*" [All Fields] OR "epidemiologic variable*" [All Fields] OR "relevant factor*"[All Fields] OR "relevant variable*" [All Fields] OR "related factor*" [All Fields] OR "related variable*" [All Fields] OR "contributing factor*"[All Fields] OR "cause*"[All Fields] OR "pathway*"[All Fields] OR "pathological mechanism*"[All Fields] OR "pathophysiology"[All Fields] OR "aetiology"[All Fields] OR "etiology"[All Fields]	
#3	"Systematic review*"[All Fields] OR "comprehensive review*"[All Fields] OR "systematic overview*"[All Fields] OR "comprehensive overview*"[All Fields] OR "meta analys*"[All Fields] OR " metaanalys*"[All Fields] OR " systematic review*"[Publication Type] OR " meta analys*"[Publication Type]	
#4	#1 AND #2 AND #3	
**The Cochrane Library Central**		
#1	All text:(ADHD or ADDH or “Attention Deficit Disorders with Hyperactivity” or “Attention Deficit Hyperactivity Disorders” or	

	"Attention Deficit Hyperactivity Disorder" or "Attention Deficit-Hyperactivity Disorder" or "Attention Deficit-Hyperactivity Disorders" or "Deficit-Hyperactivity Disorder, Attention" or "Deficit-Hyperactivity Disorders, Attention" or "Disorder, Attention Deficit-Hyperactivity" or "Disorders, Attention Deficit-Hyperactivity" or "Hyperkinetic Syndrome" or "Syndromes, Hyperkinetic" or "Attention Deficit Disorder" or "Attention Deficit Disorders" or "Deficit Disorder, Attention" or "Deficit Disorders, Attention" or "Disorder, Attention Deficit" or "Disorders, Attention Deficit" or "Brain Dysfunction, Minimal" or "Dysfunction, Minimal Brain" or "Minimal Brain Dysfunction")	
#2	All text:("Risk Factor Scores" or "Score, Risk Factor" or "Risk Scores" or "Risk Score" or "Risk Factor Score" or "Score, Risk" or "risk variable*" or predictor* or prediction or "predict* factor*" or "prognostic factor*" or "prognostic variable*" or "epidemiologic factor*" or "epidemiologic variable*" or "relevant factor*" or "relevant variable*" or "related factor*" or "related variable*" or "contributing factor*" or cause* or pathway* or "pathological mechanism*" or pathophysiology or aetiology or etiology or "Factor, Epidemiologic" or "Factors, Epidemiologic" or "Epidemiologic Determinants" or "Determinants, Epidemiologic" or "Determinant, Epidemiologic" or "Epidemiologic Determinant" or "Epidemiologic Factor" or "Factor, Risk" or "Risk Factor" or "Correlates, Health" or "Health Correlates" or "Populations at Risk" or "Population at Risk" or "Risk Factors, Social" or "Social Risk Factors" or "Risk Factor, Social" or "Factor, Social Risk" or "Factors, Social Risk" or "Social Risk Factor")	
#3	All text:("systematic review*" or "comprehensive review*" or "systematic overview*" or "comprehensive overview*" or "meta-analys*" or metaanalys* or "systematic review*")	
#4	#1 AND #2 AND #3	
	**Embase**	
#1	(ADHD or ADDH or Attention Deficit Disorders with Hyperactivity or Attention Deficit Hyperactivity Disorders or Attention Deficit Hyperactivity Disorder or Attention Deficit-Hyperactivity Disorder or Attention Deficit-Hyperactivity Disorders or Deficit-Hyperactivity Disorder, Attention or Deficit-Hyperactivity Disorders, Attention or Disorder, Attention Deficit-Hyperactivity or Disorders, Attention Deficit-Hyperactivity or Hyperkinetic Syndrome or Syndromes, Hyperkinetic or Attention Deficit Disorder or Attention Deficit Disorders or Deficit Disorder, Attention or Deficit Disorders, Attention or Disorder, Attention Deficit or Disorders, Attention Deficit or Brain Dysfunction, Minimal or Dysfunction, Minimal Brain or Minimal Brain Dysfunction).af.	
#2	(Factor, Epidemiologic or Factors, Epidemiologic or Epidemiologic	

	Determinants or Determinants, Epidemiologic or Determinant, Epidemiologic or Epidemiologic Determinant or Epidemiologic Factor or Factor, Risk or Risk Factor or Correlates, Health or Health Correlates or Populations at Risk or Population at Risk or Risk Factors, Social or Social Risk Factors or Risk Factor, Social or Factor, Social Risk or Factors, Social Risk or Social Risk Factor or Risk Factor Scores or Score, Risk Factor or Risk Scores or Risk Score or Risk Factor Score or Score, Risk or risk variable* or predictor* or prediction or predict* factor* or prognostic factor* or prognostic variable* or epidemiologic factor* or epidemiologic variable* or relevant factor* or relevant variable* or related factor* or related variable* or contributing factor* or cause* or pathway* or pathological mechanism* or pathophysiology or aetiology or etiology).af.	
#3	(systematic review* or comprehensive review* or systematic overview* or comprehensive overview* or meta-analys* or metaanalys* or systematic review*).af.	
#4	#1 AND #2 AND #3	
	**Web of science**	
#1	ALL=(ADHD or ADDH or “Attention Deficit Disorders with Hyperactivity” or “Attention Deficit Hyperactivity Disorders” or “Attention Deficit Hyperactivity Disorder” or “Attention Deficit-Hyperactivity Disorder” or “Attention Deficit-Hyperactivity Disorders” or “Deficit-Hyperactivity Disorder, Attention” or “Deficit-Hyperactivity Disorders, Attention” or “Disorder, Attention Deficit-Hyperactivity” or “Disorders, Attention Deficit-Hyperactivity” or “Hyperkinetic Syndrome” or “Syndromes, Hyperkinetic” or “Attention Deficit Disorder” or “Attention Deficit Disorders” or “Deficit Disorder, Attention” or “Deficit Disorders, Attention” or “Disorder, Attention Deficit” or “Disorders, Attention Deficit” or “Brain Dysfunction, Minimal” or “Dysfunction, Minimal Brain” or “Minimal Brain Dysfunction”)	
#2	(ALL=(“Risk Factor Scores” or “Score, Risk Factor” or “Risk Scores” or “Risk Score” or “Risk Factor Score” or “Score, Risk” or “risk variable*” or predictor* or prediction or “predict* factor*” or “prognostic factor*” or “prognostic variable*” or “epidemiologic factor*” or “epidemiologic variable*” or “relevant factor*” or “relevant variable*” or “related factor*” or “related variable*” or “contributing factor*” or cause* or pathway* or “pathological mechanism*” or pathophysiology or aetiology or etiology)) OR ALL=(“Factor, Epidemiologic” or “Factors, Epidemiologic” or “Epidemiologic Determinants” or “Determinants, Epidemiologic” or “Determinant, Epidemiologic” or “Epidemiologic Determinant” or “Epidemiologic Factor” or “Factor, Risk” or “Risk Factor” or “Correlates, Health” or “Health Correlates” or “Populations at Risk” or “Population at Risk” or	

	"Risk Factors, Social" or "Social Risk Factors" or "Risk Factor, Social" or "Factor, Social Risk" or "Factors, Social Risk" or "Social Risk Factor")	
#3	ALL=("systematic review*" or "comprehensive review*" or "systematic overview*" or "comprehensive overview*" or "meta-analys*" or metaanalys* or "systematic review*")	
#4	#1 AND #2 AND #3	
**CINAHL**		
#1	TX(ADHD or ADDH or Attention Deficit Disorders with Hyperactivity" or "Attention Deficit Hyperactivity Disorders" or "Attention Deficit Hyperactivity Disorder" or "Attention Deficit-Hyperactivity Disorder" or "Attention Deficit-Hyperactivity Disorders" or "Deficit-Hyperactivity Disorder, Attention" or "Deficit-Hyperactivity Disorders, Attention" or "Disorder, Attention Deficit-Hyperactivity" or "Disorders, Attention Deficit-Hyperactivity" or "Hyperkinetic Syndrome" or "Syndromes, Hyperkinetic" or "Attention Deficit Disorder" or "Attention Deficit Disorders" or "Deficit Disorder, Attention" or "Deficit Disorders, Attention" or "Disorder, Attention Deficit" or "Disorders, Attention Deficit" or "Brain Dysfunction, Minimal" or "Dysfunction, Minimal Brain" or "Minimal Brain Dysfunction")	
#2	TX("Factor, Epidemiologic" or "Factors, Epidemiologic" or "Epidemiologic Determinants" or Determinants, Epidemiologic" or "Determinant, Epidemiologic" or "Epidemiologic Determinant" or "Epidemiologic Factor" or "Factor, Risk" or "Risk Factor or "Correlates, Health" or "Health Correlates" or "Populations at Risk" or "Population at Risk" or "Risk Factors, Social" or "Social Risk Factors" or "Risk Factor, Social" or "Factor, Social Risk" or "Factors, Social Risk" or "Social Risk Factor" or "Risk Factor Scores" or "Score, Risk Factor" or "Risk Scores" or "Risk Score" or "Risk Factor Score" or "Score, Risk" or risk variable*" or predictor* or prediction or predict* factor* or" prognostic factor*" or "prognostic variable*" or "epidemiologic factor*" or "epidemiologic variable*" or "relevant factor*" or "relevant variable*" or "related factor*" or "related variable* or "contributing factor*" or cause* or pathway* or "pathological mechanism*" or pathophysiology or aetiology or etiology)	
#3	TX ("systematic review*" or "comprehensive review*" or "systematic overview*" or "comprehensive overview*" or "meta-analys*" or metaanalys* or "systematic review*")	
#4	#1 AND #2 AND #3	
**Scopus**		
#1	ALL(ADHD OR ADDH OR Attention Deficit Disorders with Hyperactivity" OR "Attention Deficit Hyperactivity Disorders" OR "Attention Deficit Hyperactivity Disorder" OR "Attention Deficit-Hyperactivity Disorder" OR "Attention Deficit-Hyperactivity	

	Disorders” OR “Deficit-Hyperactivity Disorder, Attention” OR “Deficit-Hyperactivity Disorders, Attention” OR “Disorder, Attention Deficit-Hyperactivity” OR “Disorders, Attention Deficit-Hyperactivity” OR “Hyperkinetic Syndrome” OR “Syndromes, Hyperkinetic” OR “Attention Deficit Disorder” OR “Attention Deficit Disorders” OR “Deficit Disorder, Attention” OR “Deficit Disorders, Attention” OR “Disorder, Attention Deficit” OR “Disorders, Attention Deficit” OR “Brain Dysfunction, Minimal” OR “Dysfunction, Minimal Brain” OR “Minimal Brain Dysfunction”)	
#2	ALL(“Factor, Epidemiologic” OR “Factors, Epidemiologic” OR “Epidemiologic Determinants” OR Determinants, Epidemiologic” OR “Determinant, Epidemiologic” OR “Epidemiologic Determinant” OR “Epidemiologic Factor” OR “Factor, Risk” OR “Risk Factor OR “Correlates, Health” OR “Health Correlates” OR “Populations at Risk” OR “Population at Risk” OR “Risk Factors, Social” OR “Social Risk Factors” OR “Risk Factor, Social” OR “Factor, Social Risk” OR “Factors, Social Risk” OR “Social Risk Factor” OR “Risk Factor Scores” OR “Score, Risk Factor” OR “Risk Scores” OR “Risk Score” OR “Risk Factor Score” OR “Score, Risk” OR risk variable*” OR predictor* OR prediction OR predict* factor* or” prognostic factor*” OR “prognostic variable*” OR “epidemiologic factor*” OR “epidemiologic variable*” OR “relevant factor*” OR “relevant variable*” OR “related factor*” OR “related variable* OR “contributing factor*” OR cause* OR pathway* OR “pathological mechanism*” OR pathophysiology OR aetiology OR etiology)	
#3	ALL(“systematic review*” OR “comprehensive review*” OR “systematic overview*” OR “comprehensive overview*” OR “meta-analys*” OR metaanalys* OR “systematic review*”)	
#4	#1 AND #2 AND #3	

The results will be reported to the citation manager: ***Endnote***. Identify and remove duplicates.

The blinded decision-making will be conducted using ***Rayyan*** software with a minimum of five researchers.

Examine the distinctions in inclusion and exclusion decisions. Address differences and create a table of inclusions with an automated rating for qualitative assessment.
