# Supplementary material for: The etiology of attention deficit disorder with hyperactivity: A protocol for an umbrella review
Source: PLoS One. 2025 Jan 24;20(1):e0318141. doi: 10.1371/journal.pone.0318141 (PMC11759348; doi:10.1371/journal.pone.0318141)
Supplement: S3 File — (PDF) [file pone.0318141.s004.pdf]

S3 File Possible conversions of some effect sizes to equivalent Ors [1]

Conversion	Justification
IRR to<br>*RR*	The following formula, straightforwardly derived from the definitions of incidence rate ratio (*IRR*) and risk ratio (*RR*), converts the former into the latter:<br>$RR = \frac{average(time_{exposed})}{average(time_{non\text{-}exposed})} \times IRR$<br>Fortunately, if the incidences are small enough, the average follow-up times are similar in exposed and non-exposed, the fraction in the left is approximately 1 and thus:<br>$RR \approx IRR$
RD to<br>*RR*	The following formula, straightforwardly derived from the definitions of risk difference (*RD*) and *RR*, converts the former into the latter:<br>$RR = 1 + \frac{1}{p_{non-exposed}} \times RD$<br>Thus, analysts might need an estimation of the probability of developing the disease (*p*) in the non-exposed.
RR to<br>*OR*	The following formula, straightforwardly derived from the definitions of *RR* and OR, converts the former into the latter:<br>$OR = \frac{1-p_{non\text{-}exposed}}{1-p_{exposed}} \times RR$<br>Fortunately, if the probabilities of developing the disease (*p*) are small enough, the fraction in the left is approximately 1, and thus:<br>$OR \approx RR$
RoM to<br>MD	The following formula, straightforwardly derived from the definitions of ratio of means (RoM) and mean difference (MD), converts the former into the latter:<br>$MD = m_{controls} \times (RoM - 1)$<br>Thus, analysts might need an estimation of the mean (*m*) in controls.
MD to<br>Glass'Δ	The following formula, straightforwardly derived from the definitions of mean difference (MD) and Glass' Δ, converts the former into the latter:<br>$\Delta = \frac{1}{s_{controls}} \times MD$<br>Thus, analysts might need an estimation of the SD (*s*) in controls.
Glass'Δ to<br>Cohen's *d*	The following formula, straightforwardly derived from the definitions of Glass'Δ and Cohen's *d*, converts the former into the latter:<br>$d = \sqrt{\frac{(n_{controls}-1)\cdot s^2_{controls}+(n_{cases}-1)\cdot s^2_{controls}}{(n[illegible]-1)\cdot s^2[illegible]+(n_{cases}-1)\cdot s^2_{cases}}} \times \Delta$<br>Fortunately, i[illegible]ses and controls are similar enough, the square root in the left is approximately 1, and thus:<br>$d \approx \Delta$
Hedge's *g* to<br>Cohen's *d*	The following formula, straightforwardly derived from the definitions of Hedge's *g* and Cohen's *d*, converts the former into the latter:<br>$d = \frac{1}{J(df)} \times g$<br>Fortunately, if the sample sizes are large enough, the small-sample correction factor (*J*) is approximately 1, the fraction in the left is approximately 1 and thus:<br>$d \approx g$
Pearson's *r* to<br>Cohen's *d*	The following standard formula[23] converts a Pearson's *r* into an approximate Cohen's *d*:<br>$d \approx \frac{2\cdot r}{\sqrt{1-r^2}}$

Reference:

1.Fusar-Poli P, Radua J. Ten simple rules for conducting umbrella reviews. Evidence-based mental health. 2018;21(3):95-100. Epub 2018/07/15. https://doi.org/10.1136/ebmental-2018-300014 PMID: 30006442.
