# Supplementary material for: The etiology of attention deficit disorder with hyperactivity: A protocol for an umbrella review
Source: PLoS One. 2025 Jan 24;20(1):e0318141. doi: 10.1371/journal.pone.0318141 (PMC11759348; doi:10.1371/journal.pone.0318141)
Supplement: S4 File — (PDF) [file pone.0318141.s005.pdf]

The operation guideline for the ecological models of health behavior

The ecological models of health behavior

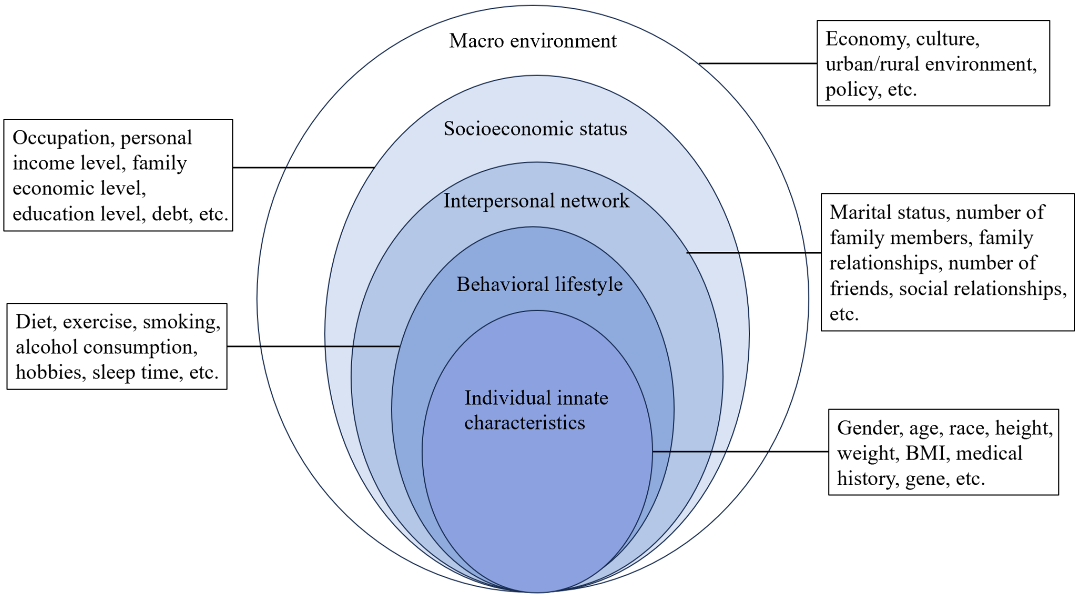

The ecological models of health behavior, which have multiple influences on specific health behaviors, including factors at the intrapersonal, interpersonal, organizational, community, and public policy levels[1], show individuals' health is affected by innate personal traits, behavior lifestyle, interpersonal network, socioeconomic status, and macro-environmental factors[2].

(1) Individual innate characteristics (including age, gender, race, height, weight, BMI, disease history, susceptible genes);

(2) Behavioral lifestyle (including diet, exercise, smoking, alcohol consumption, hobbies, sleep time, etc.);

(3) Interpersonal network (including marital status, number of family members, family relationships, number of friends, social relationships, etc.),

(4) Socio-economic status (including occupation, personal income level, family economic level, education level, debt, etc.);

(5) Macro environment (including economy, urban or rural environment, policy).

(1) Individual innate characteristics

Age: We also divided them into groups according to the World Health Organization (WHO) classification [3]: group I, young people (age <44 years); group II, middle age (age 45-59 years); group III, elderly (age 60-89 years), and group IV, extreme old age (90 years or more). For participants under the age of 44, we divided children and adolescents into ages 3-17 based on the age of the main affected population of attention deficit hyperactivity disorder (ADHD) [4].

Gender: Male or female.

Race: It is classified by white, black, and yellow people, and by nationality;

Height: It is measured in meters, weight is measured in kilograms;

BMI: The body mass index (BMI) calculation method is weight (kg)/(height (m) * height (m)); **Genetic/Medical history**: The susceptible gene or medical history reported by the healthcare institute.

(2) Behavioral lifestyle:

Diet: mainly vegetarian, mainly meat-based, and a combination of meat

and vegetables, etc;

Exercise: The frequency of exercise is calculated in hours per week; And the types of sports, such as badminton, tennis, swimming, etc;

Tobacco use: smoking, not smoking, quitting smoking, smoking after quitting, and quitting smoking many times;

Alcohol use: drinking alcohol, not drinking alcohol, quitting smoking, drinking alcohol after quitting smoking, and repeatedly quitting smoking;

Hobbies: such as intellectual games such as card games, board games, etc; Sports are the main focus, such as long-distance running, basketball, etc;

Sleep time: it's calculated based on the daily sleep time of participants.

(3) Interpersonal network

Marital status: married, single, divorced, widowed, separated.

Number of family members: current number of family members;

Family relationships: relationships with family members, good, average, not good;

Number of friends: the number of friends they have;

Other conditions at the interpersonal network level might include household activity, partners for social activity, social support, etc.

(4) Socioeconomic status:

Occupation: such as student, teacher, chef, housewife, salesperson,

engineer, etc

Personal income level: Personal monthly income, calculated in US dollars;

Family economic level: Monthly income and expenditure of the family, calculated in US dollars;

Education level: Educational qualifications are classified into primary school, secondary school, university, master's degree, doctoral degree and above;

Debt: Personal and household debt situation, calculated in US dollars;

(5) Macro environment

Economy: whether it belongs to a developed or developing country, with GDP calculated in US dollars;

Urban：

According to UN urban criteria, we define the urban area as follows:

This information is then used to classify the local administrative units (LAUs) into one of three areas[5]:

a) Densely populated area (cities): where at least 50% of the population lives in high-density clusters/urban centers. In addition, each urban center should have at least 75% of its population in a city. This ensures that all urban centers are represented by at least one city, even when this urban center represents less than 50% of the population of an LAU.

b) Intermediate density area (towns and suburbs): where less than 50% of

the population lives in rural grid cells and less than 50% live in high-density clusters;

c) Thinly populated area (rural area): where more than 50 % of the population lives in rural grid cells.

Under this method, the densely populated and intermediate density areas collectively form the city boundary.

Rural:

According to US rural criteria, we define the rural area as follows:

(1) Non-metropolitan counties;

(2) Outlying metropolitan counties with no population from an urban area of 50,000 or more people;

(3) Census tracts of at least 400 square miles in area with a population density of 35 or fewer people per square mile[6];

Policy: It refers to all regulatory legislature, such as media regulations, health sector policies, and business practices.

Other conditions at the macro environment level might include major sociopolitical shifts, such as recession, war, government collapse, safety, social climate, culture, transportation, water, sanitation, housing, etc.

Reference:

1. Sallis JF, Owen N, Fisher EB. Ecological models of health behavior. Health behavior and health education: Theory, research, and practice, 4th

ed. San Francisco, CA, US: Jossey-Bass; 2008. p. 465-85.

2. Lu J, Wang Y, Hou L, Zuo Z, Zhang N, Wei A. Multimorbidity patterns in old adults and their associated multi-layered factors: a cross-sectional study. BMC Geriatr. 2021;21(1):372. Epub 2021/06/21. https://doi.org/10.1186/s12877-021-02292-w PMID: 34147073.

3. Ahmad OB, Boschi Pinto C, Lopez A, Murray C, Lozano R, Inoue M. Ahmad OB, Boschi-Pinto C, Lopez AD, et al. 2000. Age Standardization of Rates: A New WHO Standard. GPE Discussion Paper Series: No 31, World Health.

4. Popit S, Serod K, Locatelli I, Stuhec M. Prevalence of attention-deficit hyperactivity disorder (ADHD): systematic review and meta-analysis. European psychiatry : the journal of the Association of European Psychiatrists. 2024;67(1):e68. Epub 2024/10/09. https://doi.org/10.1192/j.eurpsy.2024.1786 PMID: 39381949.

5. Nations U. What is a city. Available from: https://unhabitat.org/sites/default/files/2020/06/city_definition_what_is_a_city.pdf.

6. Administration HRS. How We Define Rural. Available from: https://www.hrsa.gov/rural-health/about-us/what-is-rural.
