# Supplementary material for: The etiology of attention deficit disorder with hyperactivity: A protocol for an umbrella review
Source: PLoS One. 2025 Jan 24;20(1):e0318141. doi: 10.1371/journal.pone.0318141 (PMC11759348; doi:10.1371/journal.pone.0318141)
Supplement: Attachment — Submitted filename: Response to Reviewers.docx [file pone.0318141.s006.docx]

Dear editor and reviewers:

On behalf of my co-authors, we appreciate editor and reviewers very much for their constructive comments and suggestions on our manuscript entitled “*The Etiology of Attention Deficit Disorder with Hyperactivity: A Protocol for an Umbrella Review*”

We have studied the comments carefully and made corrections which we hope meet with approval. The corrections are in the manuscript and the responds to the reviewers’ comments are as follows**:**

**Reviewer #1:**

(1) The retrieval strategies for each database should be elaborated in detail, rather than just providing an example of the search strategy for PubMed. This can ensure the repeatability of the retrieval process and also facilitate other researchers to evaluate the rationality of the retrieval.

Answer: Thank you for your advice. We have reset the search strategies for the other databases in S2 File.

(2) Please supplement the Sensitivity analysis. Regarding the issues of loss of original research information and subjective influence, explain how to reduce their impact and improve the reliability of research results through rigorous methodologies (such as adopting standardized data extraction and quality assessment methods, conducting sensitivity analysis, etc.) and quality assessment (such as evaluating the background and conflict of interest of the authors of systematic reviews and meta - analyses, and testing the stability of research results, etc.).

Answer: Thank you for kind advice. We will use ROBIS, AMSTAR-2, PRISMA-2020, GRADE to assess the risk of bias, methodological quality, report quality, evidence quality respectively.

We will employ the I^2^ statistic and Cochrane Q test for heterogeneity analysis, along with sensitivity analysis (utilizing the trim and fill approach and exclusively using a specific study type, such as prospective studies) to evaluate the stability of research findings. We will utilize the OR as a consolidated effect measure for conversion, as indicated in Paolo's report (S3 File). We will also examine the author's conflict of interest statement for any possible conflicts of interest.

As for the data extraction, we made changes as follows:

*Data extraction*

*“Two reviewers will independently extract data. Such as author of the study, year of publication, country of origin, kind of study (human/animal, cross-sectional, cohort, case-control, randomized controlled trial), characteristics of participants, number of cases and total participants, diagnostic criteria for ADHD, estimates of relative risk (RR), odds ratio (OR), or hazard ratio (HR) with 95% confidence intervals (CIs); Three independent reviewers will extract the quality assessment tool for heterogeneity estimation (I^2^, Cochrane's Q test), Egger's test, Begg's test, funnel plot, risk variables, and results/findings utilizing Excel 2023 software. If there is a disagreement, the final decision will be made by the third reviewer.*

*We will employ the I^2^ statistic and Cochrane Q test for heterogeneity analysis, along with sensitivity analysis (utilizing the trim and fill approach and exclusively using a specific study type, such as prospective studies) to evaluate the stability of research findings. We will utilize the OR as a consolidated effect measure for conversion, as indicated in Paolo's report (S3 File)[24]. We will utilize the quality assessment report via PRISMA-2020 to evaluate the robustness of the study findings. We will examine the author's conflict of interest statement for any possible conflicts of interest.”*

(3) Refine the classification operation of the health ecological model: Elaborate on the specific factors included in each category (macro - environment, interpersonal relationships, individual congenital characteristics, behavioral lifestyles, and socioeconomic status) in the health ecological model, and provide specific operation guidelines for classification. For example, in the macro - environment category, clarify the definition criteria of urban or rural environments (such as population density, infrastructure, etc.). At the same time, explain how to ensure the accuracy and consistency of classification during the data extraction and analysis process.

Answer: Thank you for very detailed suggestion. We researched the theory of ecological models of health behavior. Here are our changes in the following paragraph.

*“Data summary*

*We will use the ecological models of health behavior to classify the risk factors of ADHD. According to this model, risk factors can be divided into five aspects[30]: individual innate characteristics (including age, gender, race, height, weight, BMI, medical history, and susceptible genes), behavioral lifestyle (including diet, exercise, smoking, alcohol, hobbies, etc.), interpersonal network (including marital status, number of family members, number of friends, social relationships, etc.), socioeconomic status (including occupation, personal income level, family economic level, education level, debt, etc.), macro environment (including economy, urban or rural environment, policy, etc.). We have created the operation guideline of these 5 aspects (S4 File). Based on the model operation guidelines, two reviewers will classify the influencing factors. If there is a disagreement, the third reviewer will make the final decision. we will establish a classification list of ecological models of health behavior in advance, clarify the factors under each category, and make it easier to ensure the accuracy of classification when extracting data. This could help in identifying similarities and variations among significant study-included factors. We will give a detailed explanation of the outcomes of the included systematic reviews, explaining the study characteristics in relation to the intended study population, the quantity and variety of studies included, and the conclusions reached in light of the variables influencing ADHD. The differences and similarities between the included research results will be the main topic of discussion. We will use descriptive and narrative synthesis to summarize and refer to the quality of evidence.”*

(4) Please supplement the publication bias analysis.

Answer: Thank you for your careful suggestion. Here is are our correction.

*“When over 10 studies are included, we will use Egger's for publication bias analysis (P<0.1 indicates the statistical significance of publication bias).”*

**Reviewer #2:**

(1) A critical point relates to the time frame restriction. The exclusion of systematic reviews and meta-analyses published before January 2019 appears arbitrary and needs justification. While this recent cut-off would likely prevent overlap among included reviews, it might exclude valuable high-quality evidence from older, more focused reviews. The authors should either provide a clear rationale for this restriction or consider expanding the time frame. In the latter case, they would need to address how overlapping studies will be managed, for instance using the corrected covered area index or the meta-umbrella package (<https://mentalhealth.bmj.com/content/26/1/e300534>).

Answer: Thank you for your great suggestion. You are right, we have not written how to tackle overlapping issue. It’s also very important to reset the search time, just like you said it may lose some important articles or information, if we only include a period of articles. We use the corrected covered area index to fix the overlapping issue. Here are our changes.

*“Overlapping assessment*

*We will use the corrected covered area (CCA) for the assessment of overlap. We will compute CCA by constructing a graphical cross-tabulation (citation matrix) and assess the overlap rate of the original articles used in the SRMAs studies. The extent of overlap is categorized into four levels: extremely high overlap (≥15%), high overlap (11%~15%), moderate overlap (6% to 10%), and low overlap (≤ 5%). The formula for calculating CCA is as follows: CCA = N - r / [(r × c) - r]. “N” denotes the number of repeated investigations, “r” signifies the count of original studies, and “c” indicates the number of SRMAs. If the CCA is less than 10%, we deem the overlap acceptable and will proceed with direct merging for analysis. If CCA≥10%, we will prioritize selecting studies that (1) are published in the Cochrane Library, (2) are the most recent and relevant to our issue, (3) involve a larger number of participants, and (4) exhibit high quality as assessed by A Measurement Tool to Assess Systematic Reviews-2 (AMSTAR-2) and Grade of Recommendations Assessment，Development and Evaluation (GRADE) [23].”*

(2) There is also some confusion regarding the quality assessment methodology. The protocol states that GRADEpro GDT will be used to evaluate the quality of included studies. However, GRADEpro is designed to assess the certainty of evidence for specific outcomes within systematic reviews, not the methodological quality of the reviews themselves. The authors should clarify how they plan to use GRADEpro for evaluating certainty of evidence for specific outcomes, while specifying which tools will be used to assess the methodological quality of the included reviews.

Answer: Thank you for your very helpful advice. Yes, the assessment part is in mess. We followed your advice and reset our methodology. Here are our corrections:

*“****Quality assessment***

***(1) Risk of bias assessment***

*The risk of bias in systematic reviews (ROBIS) tool will be used to assess the risk of bias present in the SRMAs by two reviewers[25]. If there is a disagreement, the final decision will be made by the third reviewer. ROBIS includes three aspects: ①assessing relevance (selected according to the situation); ②Determine the level of bias risk in the process of developing system evaluations; ③Assess the risk of bias in systematic review evaluations. Each aspect of the ROBIS tool contains several landmark questions, and the answers to these questions are "yes", "possible", "no", and "possible or no information". The risk of bias in the final system evaluation is judged as low risk, high risk, or unclear. Two reviewers will independently evaluate the risk of bias quality of all included research. Should there be no agreement, the two reviewers will meet to make the final decision. In the research findings, the outcomes of the risk of bias will also be reported. When over 10 studies are included, we will use Egger's for publication bias analysis (P<0.1 indicates the statistical significance of publication bias)[26].*

***(2) Assessment of methodological quality***

*Two reviewers will independently use AMSTAR-2 to evaluate the methodological quality of the included SRMAs. This tool consists of 16 items, covering key issues included in the research, proposal, literature search, literature screening, data extraction, basic characteristics of the original research included, data analysis, and conflicts of interest, among others, including 7 key areas [27]. By evaluating the compliance of research with each criterion item, the overall quality of the system evaluation can be determined [12], which can be classified as "high" (no more than one noncritical area has defects: the system evaluation provides an accurate and comprehensive summary of outcomes); 'Medium' (defects in more than one noncritical area: systematic evaluation provides a more accurate and comprehensive summary of outcomes); Low "(with or without defects in one key area and non-key areas: the system evaluation cannot provide an accurate and comprehensive summary of outcomes); Or "extremely low" (with defects in more than one important area with or without non-important areas: the system evaluation cannot provide an accurate and comprehensive summary of outcomes based on the obtained data) at four levels.*

***(3) Assessment of report quality***

*The preferred reporting items for systematic reviews and meta-analysis 2020 (PRISMA 2020) scale will be used to evaluate the quality of SRMAs that met the inclusion criteria [28] by 2 reviewers. If there is a disagreement, the final decision will be made by the third reviewer. The PRISMA 2020 reporting standard consists of seven parts: title, abstract, introduction, methods, results, discussion, and other information, with a total of 27 items (42 sub-items). According to the completeness of the report, each item will be evaluated as a complete report (Y) with a score of 1, partial reports (PY) with a score of 0.5, and unreported (N) with a score of 0, for a total score of 42. A report with a completeness of over 80% (33-42 points) is considered "relatively complete" and rated as high-quality; A report with a completeness of over 60% (25-32 points) is considered to have some defects and is rated as of moderate quality; A report with a completeness of less than 60% (<25 points) is considered to have "relatively serious information gaps" [28, 29].*

***(4) Assessment of evidence quality***

*Two reviewers independently used the GRADE tool to evaluate the credibility of evidence in meta and systematic reviews. If there is a disagreement, the final decision will be made by the third reviewer. The GRADE system judges the increase or decrease in evidence confidence based on five factors that may lower the quality level (risk of bias, inconsistency, indirectness, inaccuracy, and publication bias) and three factors that may increase the quality level (dose-response relationship, large effect size, and negative bias evaluation of the confidence level of each outcome evidence). Ultimately, the evidence will be classified into four levels: high, moderate, low-level, and extremely low-level. And divide the epidemiological evidence for each risk factor into four categories (evidence stratification)[23]:*

*·Class I (convincing evidence): >1000 cases (or >20,000 participants for continuous outcomes), statistical significance at p<10−6 (random effects), no evidence of small-study effects and excess significance bias; 95% prediction interval excluded the null, no large heterogeneity (I2<50%);*

*·Class II (highly suggestive evidence): >1,000 cases (or >20,000 participants for continuous outcomes), statistical significance at p<10−6 (random effects) and largest study with 95%CI excluding the null value;*

*·Class III (suggestive evidence): >1000 cases (or >20,000 participants for continuous outcomes) and statistical significance at p<0.001;*

*·Class IV (weak evidence): The remaining significant associations with p<0.05;*

*·NS: non-significant: P>0.05.*

*For risk factors classified as Class I and Class II, sensitivity analysis will be conducted when they can have a significant impact on the overall results.”*

(3) Furthermore, there is ambiguity in the analysis plan. The statement suggesting that a new systematic review or meta-analysis will be conducted on risk factors ("We will do a systematic or meta-analysis on at least one clearly established risk factor...") appears inconsistent with the umbrella review methodology described elsewhere in the protocol. This needs to be clarified to avoid confusion about the intended analytical approach.

Answer: We really appreciate your detailed advice, it’s our big mistake. The right expression is “We will include any SRMAs with at least one established risk factor for ADHD, such as demographic, disease-related, or psychosocial factors.”. It should be “search SRMAs” not “do a meta-analysis”

Finally, the manuscript would benefit from English language editing. For instance, several sentences inappropriately begin with "And", and there are some typographical errors (e.g., "PROSERP" instead of "PROSPERO").

Answer: Thank you for your advice. We corrected the “PROSERP” to “PROSPERO”. We rewrote and checked our text, making sure there is no error like several sentences inappropriately begin with "And".
